# 1573. Viral Blips While on Antiretroviral Therapy are Associated with Virologic Failure

**DOI:** 10.1093/ofid/ofad500.1408

**Published:** 2023-11-27

**Authors:** Madeline Fleit, Hsing-Chuan Hsieh, Rhonda Colombo, Christina Schofield, Catherine Berjohn, Tahaniyat Lalani, Jason Blaylock, Joseph Yabes, Christie Joya, Evan Ewers, Trevor A Crowell, Xiuping Chu, Brian Agan, Anuradha Ganesan

**Affiliations:** Walter Reed National Military Medical Center, Bethesda, Maryland; Infectious Disease Clinical Research Program, Department of Preventive Medicine and Biostatistics, Uniformed Services University of the Health Sciences, Bethesda, MD, Bethesda, Maryland; Infectious Disease Clinical Research Program, USUHS, Tacoma, Washington; Madigan Army Medical Center, Tacoma, Washington; Naval Medical Center San Diego, San Diego, California; Naval Medical Center Portsmouth, Portsmouth, Virginia; Walter Reed National Military Medical Center, Bethesda, Maryland; Brooke Army Medical Center, San Antonio, Texas; Naval Medical Center Portsmouth, Portsmouth, Virginia; Fort Belvoir Community Hospital, Fort Belvoir, Virginia; Henry M. Jackson Foundation for the Advancement of Military Medicine, Bethesda, Maryland; Infectious Disease Clinical Research Program, Department of Preventive Medicine and Biostatistics, Uniformed Services University of the Health Sciences, Bethesda, MD, Bethesda, Maryland; Infectious Disease Clinical Research Program, Department of Preventive Medicine and Biostatistics, Uniformed Services University of the Health Sciences, Bethesda, MD, USA, Bethesda, Maryland; Infectious Disease Clinical Research Program, USUHS; Henry M. Jackson Foundation for the Advancement of Military Medicine Inc, Bethesda, Maryland

## Abstract

**Background:**

Blips in HIV viral load (VL) while on antiretroviral therapy (ART) are of unclear clinical significance. In this study, we examined the association between blips and virologic failure (VF).

**Methods:**

We used data from the US Military HIV Natural History Study (NHS) cohort. Included participants were diagnosed with HIV after 2006, had been on ART for ≥ 6months, and had ≥3 VLs recorded and measured using an assay with a lower limit of detection of < 50 copies/mL. VF was defined as two consecutive VLs ≥ 200 copies/mL spanning 90 days or a single VL ≥1000 copies/mL. Blips were defined as VL of 51-999 copies/mL, that were preceded and followed by a VL ≤ 50 copies/mL and were differentiated by magnitude as low-level (VL 51-199 copies/mL) or high-level (200-999 copies/mL). Detectable VLs that did not meet criteria for VF or blips were grouped as low-level viremia (LLV; 51-199 copies/mL), and higher low-level viremia (hLLV; 200-999 copies/mL). Cox proportional hazards models adjusted for demographic characteristics, time updated CD4 counts, ART, and viral load were used. Hazard ratios and 95% confidence intervals are presented.

**Figure:**
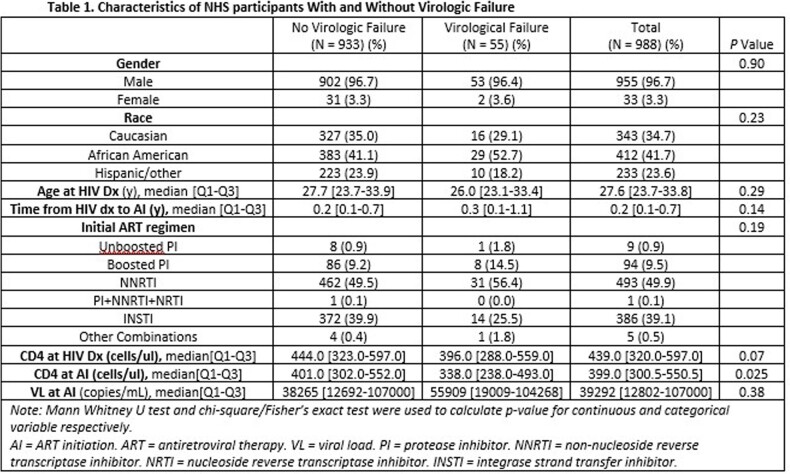

**Figure:**
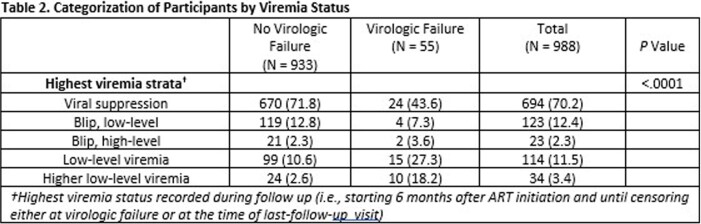

**Results:**

A total of 988 participants (median age 34.1 years, 96.7% male, 41.7% African American) were included, of which 55 (5.6%) experienced VF (Table 1). While 191 participants (19.3%) experienced blips, it was the highest VL status in 146 (14.8%) (Table 2). Having blips and low-level viremia was associated with increasing hazard of VF, graded by type and level of viremia (low-level blip 1.86 [1.10 – 3.14]; high-level blip 3.56 [1.13 – 11.18]; LLV 4.10 [3.06-5.51]; hLLV 10.51 [6.28-17.61]. Other factors associated with VF were African American race and higher VL at ART initiation. Whereas higher CD4 counts, and use of integrase strand transfer inhibitors (relative to Non-Nucleoside Reverse Transcriptase inhibitors) were protective (Table 3).

**Figure:**
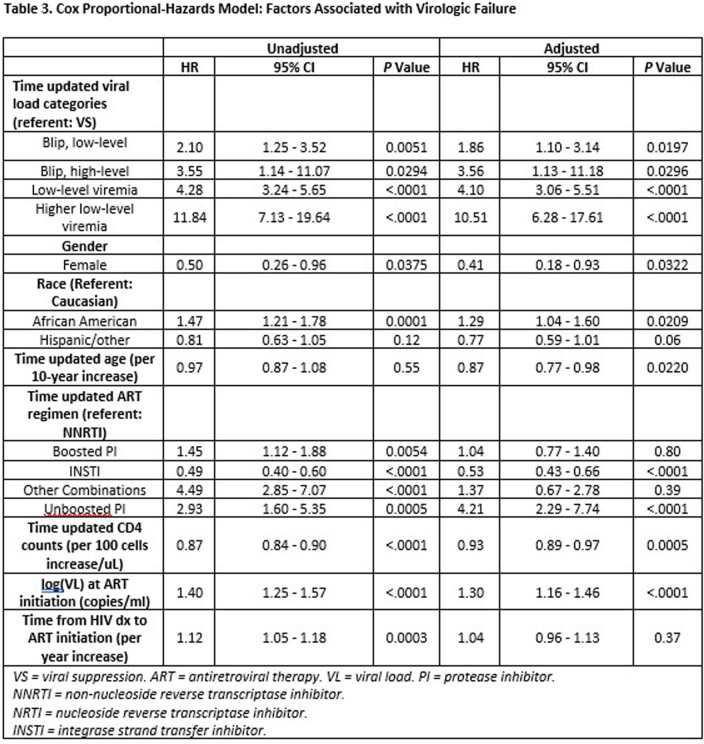

**Conclusion:**

Detectable viremia, including blips, are associated with an increased risk of VF. The magnitude of the viremia appears important with dose-response-type impact (i.e., viral loads >200 copies/mL having a greater hazard of VF). These findings suggest that individuals with viral blips at a minimum should undergo evaluation for medication adherence and may benefit from more frequent VL monitoring.

**Disclosures:**

**All Authors**: No reported disclosures

